# Comparative evaluation of warfarin utilisation in two primary healthcare clinics in the Cape Town area

**DOI:** 10.5830/CVJA-2012-072

**Published:** 2013-03

**Authors:** Xolani W Njovane, Pius S Fasinu, Bernd Rosenkranz

**Affiliations:** Division of Pharmacology, Faculty of Medicine and Health Sciences, University of Stellenbosch, Cape Town, South Africa; Division of Pharmacology, Faculty of Medicine and Health Sciences, University of Stellenbosch, Cape Town, South Africa; Division of Pharmacology, Faculty of Medicine and Health Sciences, University of Stellenbosch, Cape Town, South Africa

**Keywords:** warfarin, drug monitoring, international normalised ratio, anticoagulant, warfarinisation

## Abstract

**Background:**

Although warfarin remains the anticoagulant drug of choice in a wide range of patients, its narrow therapeutic window makes patients susceptible to a high risk of bleeding complications or failure to prevent clotting. This has necessitated therapeutic monitoring in warfarinised patients. Factors that could be responsible for the fluctuating responses to warfarin vary from pharmacogenetic to concomitant morbidity, diet and medication. In order to assess the quality of management of warfarin treatment in a local primary-care setting, the current study evaluated warfarin utilisation and monitoring records in two hospitals with different patient groups.

**Methods:**

A retrospective study was undertaken in the specialised warfarin clinics at Wesfleur and Gugulethu hospitals (Western Cape, South Africa) covering all warfarin-related therapy records over a 12-month period. Data extracted from the patients’ folders included age, gender, race, weight, address, concurrent chronic illnesses, treatment and medication, indication for warfarin and INR history.

**Results:**

A total of 119 patients’ folders were analysed. Attendance at the clinics reflects the demographics and racial distribution of the host location of the hospitals. While all the patients were maintained above the minimum international normalised ratio (INR) value of 2, about 50% had at least one record of INR above the cut-off value of 3.5. However, over a third of the patients (32.2%) had at least one record of INR greater than 3.5 in Gugulethu Hospital, compared to over half (58.3%) in Wesfleur Hospital.

In total, atrial fibrillation was the most common indication for warfarinisation while hypertension was the most common concurrent chronic condition in warfarinised patients. All patients who received quinolone antibiotics had INR values above the cut-off point of 3.5 within the same month of the initiation of antibiotic therapy, suggesting drug-induced warfarin potentiation. Other co-medications, including beta-lactam antibiotics, non-steroidal anti-inflammatory drugs (NSAIDs) and anti-ulcer drugs appeare to alter warfarin responses as measured by recorded INR values.

**Conclusion:**

The study found inter-individual variability in the response to warfarin therapy, which cut across racial classifications. It also confirms the possible influence of concomitant morbidity on patient response to anticoagulant therapy.

## Abstract

Warfarin is a racemic mixture of two optically active (R and S) isomers in roughly equal proportions, which is employed for the prevention and treatment of thrombosis signalled by atrial fibrillation, venous thromboembolism and prosthetic heart valves. Warfarin inhibits vitamin K epoxide reductase complex 1 (VKORC1), preventing the intrahepatic recycling of vitamin K epoxide to vitamin K, thus effectively supressing the vitamin K-dependent activation of clotting factors.^1^-^4^

In addition, warfarin interferes with the function of two important physiological anticoagulant proteins, C and S. S-warfarin has about five times the potency of the R-isomer with regard to vitamin K antagonism.^5^,^6^ Rapidly absorbed following oral absorption, S-warfarin undergoes CYP2C9-mediated metabolism to form 7-hydroxywarfarin, while the metabolism of the R-isomer is catalysed by CYP1A2 to 6- and 8-hydroxywarfarin, by CYP3A4 to 10-hydroxywarfarin, and by carbonyl reductases to distereo-isomeric alcohols.^7^-^9^

Warfarin has a narrow therapeutic window and to achieve treatment goals with the lowest risk of treatment failure or bleeding complications, therapeutic anticoagulation, as measured by the international normalised ratio (INR), must be achieved and sustained in patients. The dose response for warfarin is unpredictable in individual patients. It is therefore recommended that the INR is monitored daily during the initiation phase, on alternate days for a week after achieving the desired target, and once stabilised, once a month.^10^-^12^ The importance of therapeutic monitoring of warfarin is further emphasised by the fact that warfarin therapy is contraindicated in cases when INR monitoring is not feasible.^13^

Recommended therapeutic ranges of INR are 2.0–3.0 for most disease indications, and 2.0–3.5 with cardiac valve prostheses.^14^ Values outside this range may pose safety concerns. Various factors responsible for fluctuating INR in warfarin therapy include poor compliance, dosage error, concurrent illness, liver and kidney dysfunction, concomitant use of other drugs, dietary interaction, laboratory error, and ageing.^15^,^16^

Inter-individual responses to warfarin may vary due to genetic factors.^17^,^18^ The effects of concomitantly administered drugs on the pharmacokinetics of warfarin have been extensively investigated.^19^-^21^ The pharmacodynamic activity of warfarin is strongly related to the fractions of free (unbound) drug. Therefore drugs that alter the plasma protein binding of warfarin, including valproic acid and non-steroidal anti-inflammatory drugs (NSAIDs), can potentiate the anticoagulant effects of warfarin.^22^ In addition, drugs such as NSAIDs that possess antiplatelet activity can produce additive anticoagulant effects on concurrent administration with warfarin.^23^

Broad-spectrum antibacterial agents, through their effects on the vitamin K-producing gut flora, increase the effect of warfarin.^24^ Competitive substrates, inducers and inhibitors of CYP2C9 and CYP3A4 can alter warfarin plasma levels, with consequent alterations in INR.^25^ Therefore studies on utilisation and responses of warfarin in different patient groups are important to assess the causes of differences in its clinical response in a specific environment.

In South Africa, primary healthcare anticoagulation clinics play an essential role in warfarin therapy. These clinics are responsible for the education, optimisation and maintenance of anticoagulant therapy in referred patients. The initiation of anticoagulation is usually performed by the referring doctor following appropriate diagnosis and indications. The anticoagulant clinics are responsible for ensuring there are no contraindications to warfarin therapy (especially the presence of severe bleeding, first and third trimester pregnancy, and severe hepatic disorders) and ascertaining compliance to therapy.

The aim of the present study was therefore to evaluate warfarin utilisation in two primary-care anticoagulation clinics in Cape Town, Western Cape, South Africa. The study aimed at retrospective assessment of INR monitoring with consideration of possible influences of co-medication on therapy.

## Methods

A retrospective study was undertaken of all warfarin-related prescriptions in the warfarin clinics of Wesfleur and Gugulethu hospitals, covering a 12-month period between June 2008 and May 2009. Wesfleur Hospital is located in Atlantis, an area under the West Coast district municipality with a population of about 140 000 people, the majority being Coloured [the race classification was based on the national census categories, and described as black (Africans), Coloured, Indian and white]. It is a level-two facility which sees an average of 13 000 patients monthly. It runs a warfarin clinic every Friday, managed by a doctor and supported by specialist physicians at New Somerset Hospital, Cape Town, for referrals.

Gugulethu Hospital is situated in the highly populated Gugulethu Township in the City of Cape Town municipality, and is inhabited primarily by blacks. The hospital takes care of about 6 800 patients per month. The warfarin clinic is mostly managed by a nursing sister or staff nurse, who contacts a doctor if the patient’s INR results are abnormal.

Data extracted from the patient folders included age, gender, race, weight, address, concurrent chronic illnesses and medication, INR history (monthly INR levels measured in the 12-month period of the study) and indication for warfarin therapy. For the purpose of this study, a cut-off INR level of 3.5 was chosen. Patients above this limit have an increased risk of toxicity, as discussed above. Patients were assigned to the INR > 3.5 group if they had one or more INR levels above 3.5 during the course of the study.

Medications taken concurrently were pre-classified as potentially relevant or non-relevant for drug–drug interactions with warfarin using the South African Medicines Formulary (SAMF). A list of drugs taken concurrently that could result in drug–drug interactions was compiled.

Ethics approval for the project was obtained from the Health Research Ethics Committee of the University of Stellenbosch, and Wesfleur and Gugulethu Hospital managements approved this project.

MS Excel was used to capture the data and STATISTICA version 8 (data analysis software system, www.statsoft.com) (StatSoft Inc, 2008) was used for data analysis. Summary statistics was used to describe the variables. The Chi-square test was used for statistical comparison between groups. A *p*-value < 0.05 represented statistical significance in hypothesis testing.

## Results

A total of 111 patient folders were retrieved and qualified for this study after the exclusion of eight (four from each hospital) due to incomplete data. The demographic variables are summarised in Table 1. The Wesfleur Hospital had more patients (76) on anticoagulant therapy than Gugulethu (35). The racial distribution of the patients reflected the demography of the inhabitants in the hospital locations; 88.1% of the patients in Wesfleur were Coloured while all patients from Gugulethu were black.

**Table 1:** Demographic And Inr Values For Patients From Wesfleur And Gugulethu Hospitals

	*Wesfleur (n = 76)*	*Gugulethu (n = 35)*
Gender		
Male	37.5	19.4
Female	62.5	80.6
Race		
Black	5.3	100
White	5.3	0
Coloured	88.1	0
Unspecified race	1.3	0
Co-morbidities		
Diabetes	13	23
Hypertension	61	58
Arthritis	16	14
Chronic obstructive airway disease	11	6
Peptic ulcers	8	3
INR values		
INR > 3.5	58.3	32.2
Gender vs INR		
Male: INR > 3.5	51.9 (*n* = 27)	50 (*n* = 6)
Female: INR > 3.5	62.2 (*n* = 45)	28 (*n* = 25)
Age vs INR		
Patients > 40 years	86.1	58.1
Patients > 40 years: INR > 3.5	59.7 (*n* = 62)	33 (*n* = 18)
Patients < 40 years: INR > 3.5	40 (*n* = 10)	23 (*n* = 13)
Weight vs INR		
Patients > 70 kg	33.7	82.4
Patients > 70 kg: INR > 3.5	72 (*n* = 32)	35.7 (*n* = 14)
Patients < 70 kg: INR > 3.5	55 (*n* = 31)	33.3 (*n* = 3)

There was a significant variation in INR records in both hospitals. While none of the patient records showed an INR less than 2, over a third of the patients (32.2%) had at least one record of INR greater than 3.5 in Gugulethu Hospital, compared to 58.3% for Wesfleur Hospital. INR values above 3.5 generally signify high risks of bleeding. The fact that these high records were present despite monthly monitoring further underscores the importance of monitoring of warfarin therapy. It is an indication that without the hospital facility for monitoring, bleeding complications would have arisen in many of the patients.

More female patients (68%) were enrolled in the clinics than males. Considering gender and INR values, female patients’ responses to warfarin in Wesfleur Hospital suggested sensitivity, with 61% of them recording at least one INR above 3.5, compared with 23% in Gugulethu Hospital. While gender-based conclusions cannot be made based merely on this observation, several other unreported factors could account for the higher sensitivity in the female patients. This may include concomitant use of birth-control pills and differences in the use of complementary medicines or diets. Differences in body protein-to-fat ratio may also influence the effective plasma warfarin concentration in men and women, with the resultant differences in sensitivity.

## Discussion

There appeared to be differences in INR values along age and racial classification. About 64% of Coloured patients above the age of 40 years had INRs above 3.5 in Wesfleur Hospital, whereas in Gugulethu, only 33% of black patients in same age group had a record of at least one INR above 3.5. Although, no study has reported ethnic/genotype variations in warfarin response between Coloured and black people in South Africa, the body of evidence supporting genetic factors as a key influence on the response to warfarin therapy is increasing.

Scott and co-workers^26^ investigated the genetic influence on the inter-individual warfarin dose variability among various racial groups. The results revealed significant variation in the genetic expression of CYP2C9, VKORC1 and CYP4F2 in different ethnic groups. The study identified this variation as a major reason why current genotype-guided warfarin dosing algorithms in America may not yield similar results in all ethnic groups. In another study, age, body size and CYP2C9 genotype were found to be crucial determinants of warfarin dose requirements in different racial and ethnic groups.^27^

Earlier findings have shown evidence of the influence of several genes on the response to warfarin therapy, particularly the polymorphisms in CYP2C9 and VKORC1.^28^ These studies have consistently revealed that such genetic influence is less common in African–Americans compared to European–Americans and Asians.^29^ The recent report of a new genetic variant in VKORC1 among African–American populations, supported by various other warfarin pharmacogenetic studies, suggests a different warfarin maintenance dosing requirement based on genetic composition.^30^-^33^

Seventy-two per cent of patients with a body weight above 70 kg and 55% below 70 kg in Wesfleur Hospital had an INR above 3.5. In Gugulethu Hospital, 35.7% of those who weighed more than 70 kg and 33.3% of those who weighed less than 70 kg had records of an INR above 3.5. This underscores the absence of weight as a factor in the fluctuation of INR values.

High body weight is an important risk factor of the indications for warfarin therapy in patients with cardiovascular disorders. The pharmacokinetic disposition and activity of warfarin may be influenced by body weight. The effects of weight may therefore be a necessary consideration in the attainment of a stable INR in warfarinised patients.

When the concurrent chronic diseases of patients attending the warfarin clinics were evaluated, hypertension was the most common disease in both hospitals (57.9% in Wesfleur and 51.4% in Gugulethu Hospital). Hypertension is a chronic lifestyle-related disease with body weight and genetic factors as main risk factors. The maintenance of INR values within an acceptable therapeutic range will be particularly taxing in patients with hypertension and other cardiovascular disorders. In addition to the effects of the cardiovascular medications, fluctuations in cardiac function, such as cardiac output and peripheral vascular resistance may play a significant role in the body distribution of and sensitivity to warfarin.

Hypertension may therefore play a significant role in the warfarin response in these patients. Long-standing hypertension is associated with complications such as atrial fibrillation. This is reflected in the data, as atrial fibrillation was the most common clinical indication for the initiation of warfarin therapy at Wesfleur Hospital (47%), and the second most common indication (39%) in Gugulethu Hospital (Table 2).

**Table 2:** Indications For Warfarin Therapy For Patients From Wesfleur And Gugulethu Hospitals (Some Patients Had Multiple Indications)

*Indication*	*Wesfleur (%)*	*Gugulethu (%)*
Myocardial infarction	0.3	0
Valve replacement	31	39
Mixed valve disease	35	42
Atrial fibrillation	47	39
Thrombosis/embolism	24	6

Concurrent medications that were commonly prescribed for patients on warfarin therapy were antibiotics, especially fluoroquinolones, beta-lactams and metronidazole; non-steroidal anti-inflammatory drugs; paracetamol; and anti-ulcer drugs (Table 3). Quinolones were prescribed at only Wesfleur Hospital, and an elevated INR above the cut-off value of 3.5 was recorded in all the patients concerned in the month that these drugs were taken concurrently.

**Table 3:** Comparison Of Data For Concurrent Medication Prescribed Vs INR In Patients At For Patients From Wesfleur And Gugulethu Hospitals

	*Wesfleur*		*Gugulethu*	
*Medications*	*INR > 3.5*	*INR < 3.5*	*INR > 3.5*	*INR < 3.5*
Quinolones	7	0	0	0
Beta-lactams	9	3	1	1
Metronidazole	3	1	1	1
NSAIDs	1	1	1	0
Paracetamol	6	8	3	0
Anti-ulcer drugs	3	3	0	1

A similar occurrence was observed with beta-lactams in both hospitals; 71.4% of patients treated with these antibiotics had INR values above 3.5. The effects of broad-spectrum antibiotics on the vitamin K-producing gastrointestinal microflora can potentiate the anticoagulant effects of warfarin. This may explain the observation of elevated INR in warfarinised patients on concomitant antibiotic therapy. In addition, quinolones are substrates and inhibitors of various CYP isoforms including CYP3A4.^34^ Pharmacokinetic interaction at the metabolic level with these antibiotics can inhibit warfarin metabolism, leading to increased INR, as observed in this study.

Various other studies have reported an interaction between warfarin and the quinolones, metronidazole and other antibacterial drugs, leading to bleeding complications. Therefore it is reasonable to infer that drug–drug interactions could have been responsible for the observed increase in INR in the month of concurrent administration with warfarin.

The observations presented in this study reflect the complexity of the many factors to be considered in warfarin therapy. A comprehensive overview of such considerations by McMillin and co-workers^35^ concluded that pharmacogenetic testing is important in personalised warfarin therapy. While new anticoagulant drugs are entering the drug market and clinical practice, warfarin will continue to be the reference oral anticoagulant.

This is more so in Africa, where cost and accessibility are important considerations. Therefore, studies that will add to current knowledge and improve warfarin therapy among African populations will benefit clinicians, patients, researchers and policy makers in the health sector.

## Conclusions

This study confirmed the variability in patient response to warfarin therapy, with race, gender, weight, and concurrent morbidity and medications as some of the important factors. Over a third of the patients had at least one record of an INR above 3.5 in Gugulethu Hospital, compared to over half in Wesfleur Hospital. Differences in the control of INR values were observed with race, weight and age.

Warfarin remains the oral anticoagulant of choice in South Africa. Its use should be closely monitored. Health practitioners must be aware of the various factors responsible for variations in inter-individual responses to warfarin therapy. INR values should be monitored frequently and closely in high-risk patients, including those on co-medications and with cardiovascular co-morbidities.
